# Comparison of pigment epithelium detachment composition indices between neovascular age-related macular degeneration and polypoidal choroidal vasculopathy

**DOI:** 10.1186/s40942-023-00512-6

**Published:** 2024-02-15

**Authors:** Stavan V. Shah, Sumit Randhir Singh, Amrish Selvam, Sanjana Harihar, Yash Parmar, Rubble Mangla, Supriya Arora, Kiran K. Vupparaboina, Ramesh Venkatesh, Jay Chhablani

**Affiliations:** 1 University of Pittsburgh School of Medicine, Pittsburgh, PA USA; 2 Sharp Sight Eye Hospital, Aadya Heights, Ashiana Modh, Ashiana - Digha Rd, Patna, Bihar 800025 India; 3 University of Pittsburgh, Pittsburgh, PA USA; 4 Department of Retina-Vitreous, Narayana Nethralaya, Bangalore, Karnataka India; 5 Bahamas Vision Center and Princess Margaret Hospital, Nassau, Bahamas; 6 Department of Ophthalmology, UPMC, Pittsburgh, PA USA; 7 Department of Ophthalmology, University of Pittsburgh School of Medicine, Pittsburgh, PA USA

**Keywords:** Pigment epithelium detachment composition index, Neovascular age-related macular degeneration, Polypoidal choroidal vasculopathy, Optical coherence tomography, Anti-vascular endothelial growth factors

## Abstract

**Purpose:**

To compare changes in the fibrous component of pigment epithelium detachment composition indices (PEDCI-F) in neovascular age-related macular degeneration (n-AMD) and polypoidal choroidal vasculopathy (PCV) over 12 months.

**Methods:**

This was a retrospective chart review of treatment-naïve n-AMD and PCV eyes treated with anti-vascular endothelial growth factor (anti-VEGF) agents. Optical coherence tomography (OCT) images were recorded at baseline and at 3, 6, and 12 months. OCT images were processed by filtering followed by pigment epithelium detachment (PED) segmentation and analysis of PED lesion heterogeneity based on the composition (PEDCI-F).

**Results:**

A total of 74 eyes with n-AMD (36) and PCV (38) were included. Overall, PEDCI-F increased minimally in both n-AMD and PCV groups (both p > 0.05). The majority, i.e., 58.3% and 60.5%, of n-AMD and PCV eyes, respectively, showed an increase in PEDCI-F at 12 months. An increase in PEDCI-F was associated with improved BCVA logMAR (n-AMD, r = -0.79; p < 0.001 and PCV, r = − 0.06; p = 0.74) and the need for fewer anti-VEGF injections (n-AMD, r = − 0.53; p < 0.001 and PCV, r = − 0.09; p = 0.58).

**Conclusion:**

PEDCI-F increases in the majority of eyes with n-AMD and PCV through 12 months following treatment with anti-VEGF injections. This group had better visual acuity compared to the other subset with reduction in PEDCI-F requiring more anti-VEGF injections and worse visual acuity, possibly due to fibrovascular PED (FVPED) collapse and atrophy or a relative increase in other PEDCI constituents at 12 months.

## Introduction

Neovascular age-related macular degeneration (n-AMD), a subtype of AMD, is characterized by neovascularization below and/or above the retinal pigment epithelium (RPE) [1]. Fibrovascular pigment epithelium detachment (FVPED), a morphologic variant of n-AMD, also known as type 1 choroidal neovascularization (CNV), is reserved for CNV located below the RPE and carries a worse visual prognosis than CNV located above the RPE [2, 3]. Polypoidal choroidal vasculopathy (PCV), commonly considered a variant of type 1 AMD, i.e., aneurysmal type 1 CNV, is known to have a different clinical presentation with tall, notched PED, a higher incidence of sub-RPE, subretinal, intraretinal and vitreous hemorrhage and exudative complications, thereby leading to a worse visual prognosis than typical type 1 CNV [4]. PED in PCV eyes are morphologically different from n-AMD eyes and have few intrinsic differences compared to n-AMD eyes. The presence of multiple/sharp-peaked/notched/hemorrhagic PED, sub-RPE ring-like lesions, and complex structures on en face OCT are more commonly associated with PCV [5, 6].

FVPED are essentially composed of neovascular tissue, serous fluid, sub-RPE heme, and fibrous tissue [7–9]. These are known to be less responsive to treatment and therefore worse visual acuity [8, 10]. The advent of high-resolution spectral domain and swept source optical coherence tomography (OCT) has facilitated the identification of FVPED components to a certain extent. Serous and hematogenous areas are relatively easier to delineate based on the homogeneity and reflectivity pattern, whereas neovascular components are difficult to segregate based only on OCT appearance. Variability in the clinical course, disease resolution and visual prognosis of FVPED may be related to the relative distribution and changes in the constituents of FVPED [11].

We recently published the methodology and analysis of pigment epithelium detachment composition indices (PEDCI) using our automated algorithm based on the heterogeneity of the PED composition [12]. In the current work, we aimed to study PEDCI (fibrous component, PEDCI-F) changes in eyes with n-AMD and PCV treated with anti-vascular endothelial growth factors (anti-VEGF) for 12 months.

## Methods

This was a two-center (University of Pittsburgh School of Medicine, Pittsburgh, PA, United States and Narayana Nethralaya, Bangalore, Karnataka, India), retrospective chart review of treatment-naïve n-AMD and PCV patients with a minimum follow-up of 12 months. All patients were undergoing anti-VEGF monotherapy with visits recorded at 0, 3, 6, and 12 months. All patients with a history of ocular surgery, preexisting ocular disease, etiology of choroidal neovascularization other than AMD/PCV, any prior history of uveitis, glaucoma, media opacities precluding fundus view, less than 12 months of follow-up data or poor-quality OCT scans preventing PED analysis were excluded from the analysis. Patients with large hemorrhagic PED or those requiring surgical intervention due to breakthrough vitreous hemorrhage were not considered for the study.

The local institutional review board approved the study, which conformed to the tenets of the Declaration of Helsinki. All study patients signed an informed consent form regarding treatment and participation in clinical research as part of the study.

Demographic parameters included age, sex, any systemic or ocular comorbidity and treatment (both medical and surgical) history. Baseline and follow-up examinations included best-corrected visual acuity (BCVA) measured in logarithm of minimum angle of resolution (logMAR), anterior and posterior segment examination using slit lamp biomicroscopy and intraocular pressure measurement. Baseline and registered follow-up OCT images were obtained on a Heidelberg Spectralis (Heidelberg Engineering, Heidelberg, Germany). OCT measurements at each visit included central macular thickness (CMT) and subfoveal choroidal thickness (SFCT) on a single line scan passing through the fovea. Patients were initially treated with a loading dose of 3 anti-VEGF injections followed by a pro-re-nata (PRN) regimen during subsequent visits. The choice of anti-VEGF injection was based on the discretion of the treating physician.

Preprocessing steps, i.e., linear normalization and shadow compensation, were part of the standard protocol used to analyze OCT scans. This was based on our previously published algorithm. Then, PED segmentation was performed to mark the outer boundary, and further analysis was performed to classify PED into 3 components based on the heterogeneous composition, i.e., serous, neovascular and fibrous (PEDCI S, N and F, respectively). This was done by computing the scarring likelihood metric based on multiple filtering steps. Here, we used PEDCI-F, the ratio of fibrous tissue area to the total PED lesion [12].

### Statistical analysis

Data are presented as the mean ± standard deviation or median (interquartile range, IQR). All statistical analyses were performed using Statistical Package for the Social Sciences (SPSS version 24, New York, USA). The Kolmogorov‒Smirnov test was used to assess the normality distribution. Nonparametric tests were employed because the data were not normally distributed. The Friedman test was used to compare the parameters at different time periods. In case of statistically significant results, pairwise comparisons were performed using the Wilcoxon signed-rank test. A P value ≤ 0.05 was considered statistically significant.

## Results

A total of 74 eyes, including 36 eyes of 34 patients and 38 eyes of 35 patients with n-AMD and PCV, respectively, were analyzed. The study cohort had a preponderance of females in the n-AMD group and included 8 males (23.5%) and 26 females (76.5%), whereas the PCV group included 19 males (54.3%) and 16 females (45.7%).

### Best corrected visual acuity (BCVA)

**AMD**: BCVA was 0.44 ± 0.26 logMAR (mean ± SD) at baseline with a median (IQR) of 0.41 (0.25 to 0.57). There was minimal worsening of BCVA (mean ± SD: 0.51 ± 0.26) at the last follow-up; however, the change in BCVA was not statistically significant through 12 months (p = 0.19).

#### PCV

Baseline BCVA was 0.63 ± 0.62 (mean ± SD), with median (IQR) values of 0.5 (0.2 to 0.75). Through 12 months, BCVA logMAR improved to 0.43 ± 0.58 (mean ± SD). The Friedman test comparing BCVA at baseline and at 3, 6 and 12 months showed a statistically significant change (p < 0.001). Pairwise comparison using the Wilcoxon signed-rank test showed a significant difference between baseline and 3 months (p = 0.005) and baseline and 12 months (p = 0.002).

### Pigment epithelium detachment composition index – fibrous component (PEDCI-F)

#### n-AMD

The comparison of the PEDCI-F scores at different time points (baseline, 3, 6, and 12 months) using the Friedman test was not statistically significant (p = 0.13). The PEDCI-F score at baseline was 0.32 ± 0.28 (mean ± SD) (median, 0.27; IQR, 0.05 to 0.55), which increased marginally to 0.35 ± 0.31 (mean ± SD) at 12 months (median, 0.08; IQR, 0.15 to 0.64). The PEDCI-F increased by 0.24 ± 0.24 (mean ± SD) in 21/36 eyes (58.3%), whereas the remaining 15/36 eyes (41.7%) had a reduction in the PEDCI (mean ± SD: 0.25 ± 0.20) through 12 months.

#### PCV

**Fig. 1:**
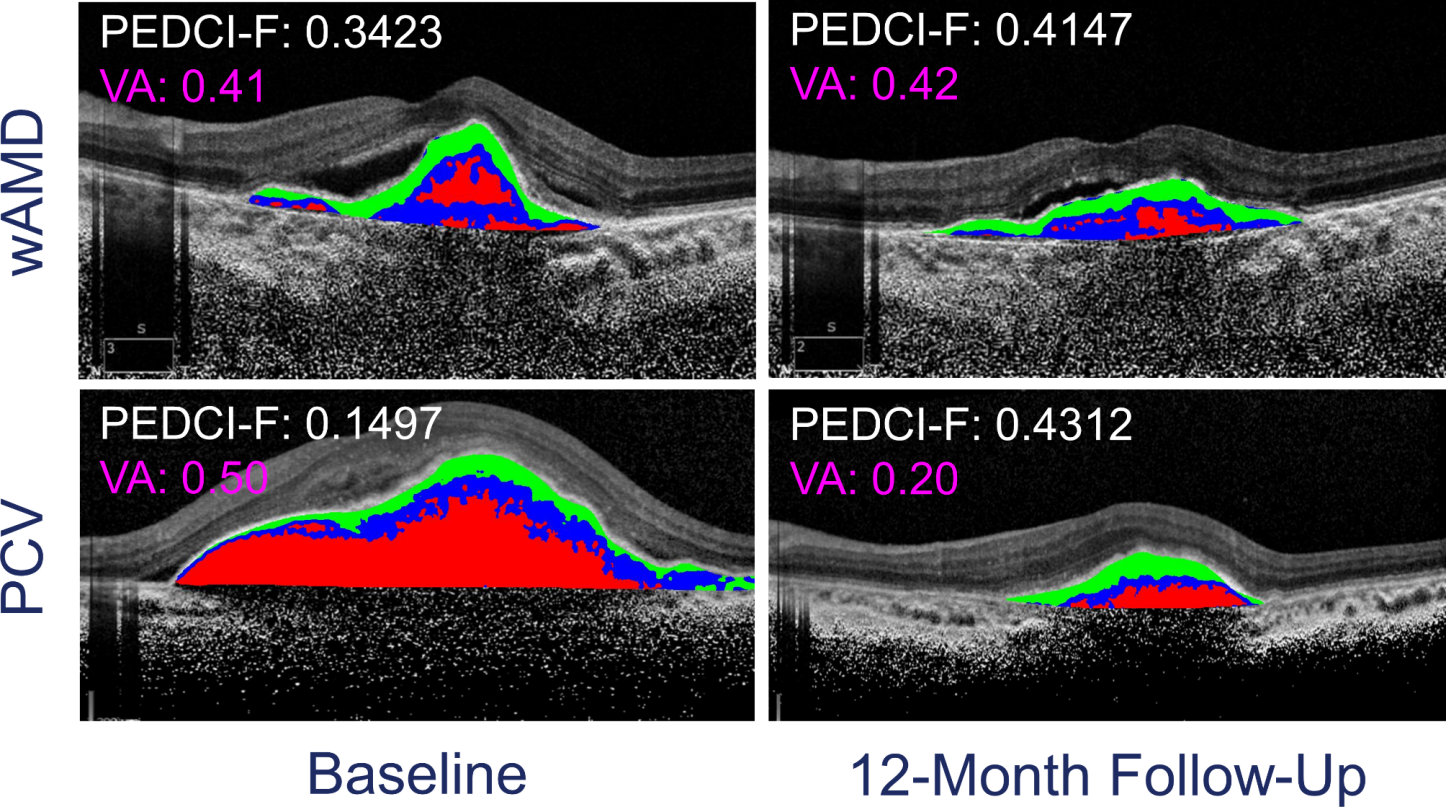
Heatmaps demonstrating the scoring of fibrovascular pigment epithelium detachment (FV-PED) lesions with PED composition indices (PEDCI), where green represents the fibrous component (PEDCI-F), blue represents the neovascular component, and red is the serous component of the lesion. Numerical values of PEDCI-F (white) and best corrected visual acuity (BCVA, logarithm of minimum angle of resolution logMAR; pink) are overlaid

PEDCI-F at baseline was 0.24 ± 0.27 (median, 0.12; IQR, 0.05 to 0.35), which increased to 0.38 ± 0.31 at 12 months (median, 0.29; IQR, 0.12 to 0.55). The change in the PEDCI-F score over 12 months was not statistically significant (p = 0.19). The majority of PCV eyes (23/38; 60.5%) had increased PEDCI-F scores at the final visit compared to the baseline visit, with a mean (± SD) increase of 0.36 ± 0.33. On the other hand, 15/38 (39.5%) eyes showed reduced PEDCI-F (0.20 ± 0.21) at the last visit. Representative cases of both AMD and PCV are shown in Fig. 1.

### Central macular thickness (CMT)

#### n-AMD

CMT showed a significant reduction from 321.7 ± 126.5 μm to 227.3 ± 95.6 μm (p < 0.001). Pairwise comparisons using Wilcoxon signed-rank test with Bonferroni adjustment showed significant differences between baseline and all 3 follow-up visits (3, 6, and 12 months) (all p values < 0.001).

#### PCV

There was a statistically significant reduction in CMT from 411.1 ± 175.1 μm at baseline to 296.0 ± 93.4 μm at 12 months (p < 0.001). Pairwise comparisons between baseline and 3, 6, and 12 months showed significant differences between baseline − 3 months (p < 0.001), baseline − 6 months (p = 0.002) and baseline − 12 months (p < 0.001).

### Subfoveal choroidal thickness (SFCT)

#### nAMD

SFCT was reduced marginally from 191.4 ± 56.7 μm (median, 190.5 μm; IQR, 155.0 to 235.8 μm) at baseline to 190.4 ± 52.4 (median, 198.0 μm; IQR, 149.5 to 231.8 μm) (p = 0.71).

#### PCV

At baseline, eyes with PCV had a thicker choroid (mean ± SD: 335.8 ± 158.9 μm) than n-AMD eyes (191.4 ± 56.7 μm). SFCT was reduced from 335.8 ± 158.9 μm (median, 325.5 μm; IQR, 274.0 to 361.3 μm) at baseline to 289.5 ± 117.0 μm through 12 months (median, 301.5 μm; IQR, 192.8 to 350.2 μm; p = 0.30).

### Number of anti-VEGF injections

Eyes with n-AMD received 8.8 ± 1.9 anti-VEGF injections over 12 months. Among these, 60.4% and 39.6% of total injections were bevacizumab (Avastin®, Genentech, South San Francisco, USA), and aflibercept (Eylea®, Regeneron, Tarrytown, New York, USA) respectively. On the other hand, PCV eyes were treated with a mean ± SD of 8.1 ± 4.2 anti-VEGF injections during the follow-up, which was not significantly different from n-AMD eyes (p = 0.77). Among these, 59.2% and 40.8% anti-VEGF injections were avastin and eylea respectively.

Correlation analysis between BCVA (logMAR) and PEDCI-F showed a strong negative correlation (r = -0.79; p < 0.001) in n-AMD, whereas the negative correlation was weak in PCV eyes (r = -0.06; p = 0.74). Similarly, the strength of the association between the number of anti-VEGF injections and PEDCI-F was moderate, with a negative correlation (r = -0.53; p < 0.001) in n-AMD eyes. On the other hand, PCV eyes failed to show a significant correlation between the number of anti-VEGF injections and PEDCI-F (r = -0.09; p = 0.58).

## Discussion

We report the 12-month outcomes of PEDCI-F and other OCT-based parameters in eyes with n-AMD and PCV. PCV eyes showed a significant change in BCVA from 0.63 ± 0.62 at baseline to 0.43 ± 0.58 at 12 months, whereas n-AMD eyes had nonsignificant minimal worsening from 0.44 ± 0.26 to 0.51 ± 0.26. PEDCI-F increased marginally through 12 months in both groups, i.e., from 0.32 ± 0.28 (mean ± SD) to 0.35 ± 0.31 in the n-AMD group, while PEDCI-F in the PCV group increased from 0.24 ± 0.27 to 0.38 ± 0.31 at the final visit.

Although the majority of eyes in both groups (58.3% and 60.5% in n-AMD and PCV, respectively) showed an increase in PEDCI-F at 12 months, a significant number of eyes, 41.7% (n-AMD) and 39.5% (PCV), were noted to have reduced PEDCI-F at the last visit. Increased PEDCI-F may be a sign of maturation of the neovascular complex, and therefore, the disease may stabilize with fewer anti-VEGF agents. Conversely, eyes with reduced PEDCI-F through 12 months needed a higher number of anti-VEGF injections in both the n-AMD and PCV groups; however, moderate strength was seen in n-AMD eyes only (r = − 0.53; p < 0.001). Moreover, eyes with worse visual acuity also had reduced PEDCI-F scores during follow-up.

Both n-AMD and PCV eyes post treatment with anti-VEGF injections are known to develop FVPED regression followed by collapse and ultimately atrophy [9, 11]. Therefore, not all patients may manifest an increase in PEDCI-F with time, whereas a significant proportion also develop atrophy and therefore a reduction in FVPED volume and reduced scarring. The relative increase in the area of other components, i.e., neovascular and serous, also needs to be considered to explain the relative reduction in PEDCI-F.

Both n-AMD and PCV are characterized by fibrovascular PED, and differences are related to PED height, notching, and the presence of polyps with or without branching vascular networks [10, 13]. FV-PED is a heterogeneous description and may be further subdivided into serous, neovascular and fibrous components [11]. Among these, serous and neovascular components are possibly related to active disease, whereas fibrous components (PEDCI-F) may develop or increase later in the disease course. This may be associated with a worse visual prognosis.

As expected, there was a significant reduction in CMT in both the AMD and PCV groups. Although the comparative reduction in SFCT was greater in PCV eyes (46.3 μm) than in n-AMD eyes (1.0 μm), SFCT readings in none of the groups reached statistical significance.

This study has a few limitations. The effectiveness of different anti-VEGF injections on PEDCI-F was not evaluated. Moreover, the number of patients and follow-up duration were limited in each group. PEDCIs are defined based on the pixel analysis and scarring likelihood metric of each pixel. This metric is arbitrarily defined, and therefore, the differentiation of neovascular and fibrous may be erroneous. n-AMD and PCV may show regression of PED with resultant atrophy on treatment. The average capillary size ranges from 2.5 to 10 μm and is broadly similar to the axial and transverse resolution of OCT scans, suggesting that vessels in FVPED may be few pixels in size; therefore, correct characterization with current OCT technology has inherent limitations [12].

To conclude, the majority of eyes with n-AMD and PCV are associated with an increase in PEDCI-F over time despite treatment with anti-VEGF injection. However, a large subset in both groups also showed reduced PEDCI-F over 12 months, possibly due to FVPED collapse and atrophy or a relative increase in other constituents. These eyes need additional anti-VEGF agents and are associated with a worse visual prognosis. Although automated quantifiable analysis of PED constituents, especially the PEDCI-F, is possible, the utility of the PEDCI-F as a surrogate marker of disease activity and prognosis needs further validation in a large cohort with a longer duration of follow-up.

## Data Availability

Data is available from the corresponding author upon reasonable request.
